# JAK inhibitor blocks COVID-19 cytokine–induced JAK/STAT/APOL1 signaling in glomerular cells and podocytopathy in human kidney organoids

**DOI:** 10.1172/jci.insight.157432

**Published:** 2022-06-08

**Authors:** Sarah E. Nystrom, Guojie Li, Somenath Datta, Karen L. Soldano, Daniel Silas, Astrid Weins, Gentzon Hall, David B. Thomas, Opeyemi A. Olabisi

**Affiliations:** 1 Division of Nephrology, Duke Molecular Physiology Institute, Duke University School of Medicine, Durham, North Carolina, USA.; 2 Department of Pathology, Brigham and Women’s Hospital, Harvard Medical School, Boston, Massachusetts, USA.; 3 NEPHROCOR, a division of Bostwick Laboratories, Memphis, Tennessee, USA.

**Keywords:** Cell Biology, Nephrology, Cytokines, Endothelial cells, iPS cells

## Abstract

COVID-19 infection causes collapse of glomerular capillaries and loss of podocytes, culminating in a severe kidney disease called COVID-19–associated nephropathy (COVAN). The underlying mechanism of COVAN is unknown. We hypothesized that cytokines induced by COVID-19 trigger expression of pathogenic APOL1 via JAK/STAT signaling, resulting in podocyte loss and COVAN phenotype. Here, based on 9 biopsy-proven COVAN cases, we demonstrated for the first time, to the best of our knowledge, that APOL1 protein was abundantly expressed in podocytes and glomerular endothelial cells (GECs) of COVAN kidneys but not in controls. Moreover, a majority of patients with COVAN carried 2 *APOL1* risk alleles. We show that recombinant cytokines induced by SARS-CoV-2 acted synergistically to drive *APOL1* expression through the JAK/STAT pathway in primary human podocytes, GECs, and kidney micro-organoids derived from a carrier of 2 *APOL1* risk alleles, but expression was blocked by a JAK1/2 inhibitor, baricitinib. We demonstrate that cytokine-induced JAK/STAT/APOL1 signaling reduced the viability of kidney organoid podocytes but was rescued by baricitinib. Together, our results support the conclusion that COVID-19–induced cytokines are sufficient to drive COVAN-associated podocytopathy via JAK/STAT/APOL1 signaling and that JAK inhibitors could block this pathogenic process. These findings suggest JAK inhibitors may have therapeutic benefits for managing cytokine-induced, APOL1-mediated podocytopathy.

## Graphical abstract

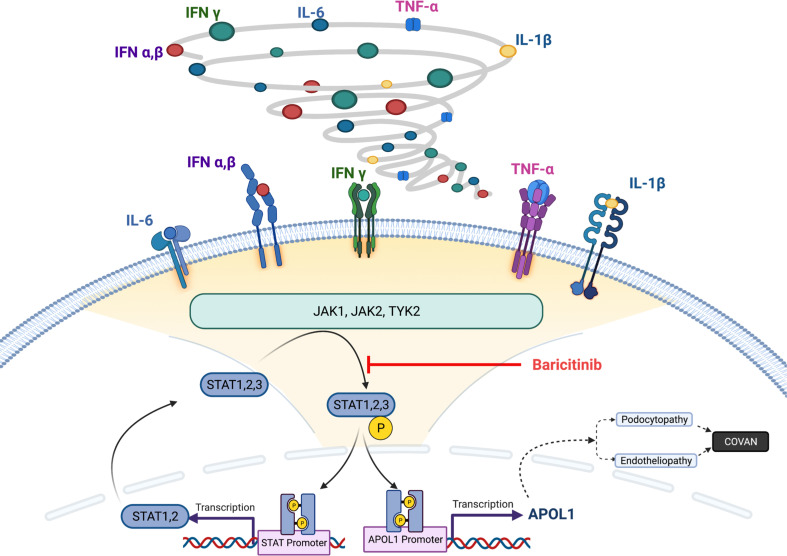

## Introduction

Kidney failure is a devastating complication of COVID-19 infection. Up to 50% of inpatient and 70% of intensive care unit COVID-19 admissions are complicated by acute kidney injury (AKI), which, in turn, increases mortality risk by 30% to 50% (1, 2). A kidney biopsy case series revealed that collapsing glomerulopathy is the most common histopathologic diagnosis in COVID-19–associated AKI (3). A distinctive feature of COVID-19–associated nephropathy (COVAN; also known as COVID-19–associated collapsing glomerulopathy) is its near-exclusive predilection for Black people who carry 2 risk alleles of apolipoprotein L1 (*APOL1*) (3, 4). The 2 risk alleles (named G1 and G2) emerged as coding variants in the *APOL1* gene and confer protection against African trypanosomiasis. However, carriage of G1G1, G2G2, or G1G2 (which are high-risk genotypes) increases the risk of a spectrum of kidney diseases and explains much of the excess risk of nondiabetic kidney disease among Black people (5–8). An estimated 13% of Black Americans carry high-risk *APOL1* genotypes (7). During the COVID-19 pandemic, researchers found that a remarkable 92% of biopsy-proven COVAN cases were in carriers of high-risk *APOL1* genotypes, 61% of whom required dialysis at presentation (3, 9). These findings establish *APOL1* variants as major contributors to the racial disparity in COVID-19 health outcomes. Despite this impressive association, the cellular mechanism that connects high-risk *APOL1* genotypes to SARS-CoV-2 infection and pathogenesis of collapsing glomerulopathy of COVAN remains unknown.

The strong epidemiologic association between high-risk *APOL1* genotype and COVAN has led to the hypothesis that COVID-19–induced expression of *APOL1* G1 or G2 in podocytes and glomerular endothelial cells (GECs) — the kidney cells affected in collapsing glomerulopathy — drives pathogenesis of COVAN. This hypothesis was supported by recent reports that transgenic overexpression of *APOL1* risk alleles in mouse podocytes or GECs caused podocytopathy, endotheliopathy, glomerulopathy, and clinical manifestations of kidney failure (10–14). These murine disease models suggest that the mechanism that underlies COVID-19–induced *APOL1* expression would be a potential therapeutic target for COVAN. However, there are 2 important unknowns: (a) It is unknown whether *APOL1* protein expression is upregulated in glomeruli of patients with COVAN, and (b) it is unknown whether SARS-CoV-2 induces *APOL1* expression directly by viral infection of kidney cells or indirectly via the effects of SARS-CoV-2–induced cytokine storm.

That SARS-CoV-2 has not been detected in kidney biopsy specimens of patients with COVAN provides indirect support for the hypothesis that COVAN likely results from the effects of cytokine storm rather than from direct viral infection of kidney parenchyma. The occasional detection of SARS-CoV-2 viral particles has been in autopsy kidney specimens in which the confounding effect of tissue autolysis could not be excluded (3, 4, 15, 16). Several inflammatory cytokines and chemokines have been noted to be upregulated in the sera of patients with COVID-19 and/or COVAN (4, 17, 18). This list includes cytokines such as IFN-α, IFN-β, IFN-γ, and TNF, which are upregulate *APOL1* expression. However, the list also includes several inflammatory cytokines that are robustly upregulated by COVID-19 infection. It is unknown whether these cytokines have additive, synergistic, or antagonistic effects on *APOL1* expression and associated podocytopathy.

In the present study, we addressed these knowledge gaps by using kidney biopsy specimens obtained from 9 patients with COVAN and 2 human control kidneys to investigate whether and where *APOL1* protein is expressed in COVAN and control kidneys. We profiled 18 COVID-19–induced cytokines to identify 8 cytokines that were sufficient, in the absence of SARS-CoV-2, to synergistically induce *APOL1* expression in primary human glomerular cells and cause podocytopathy in human kidney micro-organoids. To our knowledge, this study not only offers the first proof in a human-derived experimental model that COVID-19 cytokine storm induces *APOL1* expression and podocytopathy but also identifies the common signaling pathway that mediates the pathogenic effects. This study has implications that could influence strategies for screening and treating COVAN in Black and Hispanic patients. It raises a question about safety of supplemental IFNs as COVID-19 therapy in Black and Hispanic individuals who carry high-risk *APOL1* genotypes (19, 20).

## Results

### APOL1 expression is upregulated in podocytes and GECs of patients with COVAN.

**Figure 1:**
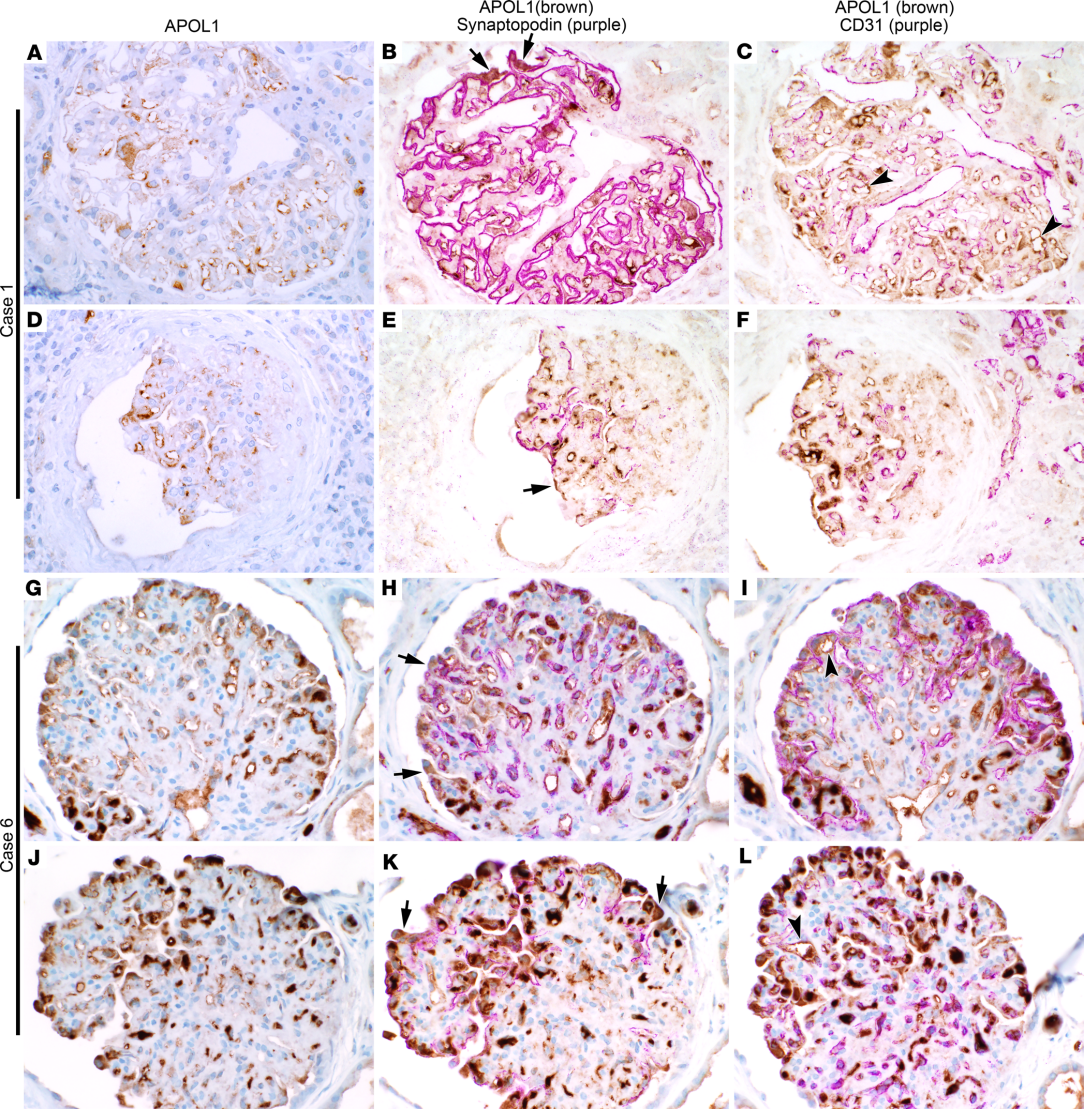
APOL1 expression is upregulated in podocytes and GECs of patients with COVAN. (**A**–**L**) IHC for APOL1 in biopsy tissue from (**A**–**F**) patient 1 and (**G**–**L**) patient 6. Original magnification, 40×. (**B**, **E**, **H**, and **K**) Co-staining of APOL1 with podocyte marker synaptopodin. (**C**, **F**, **I**, and **L**) Co-staining of APOL1 with endothelial marker CD31. Arrow represents a podocyte. Arrowhead represents a GEC.

To investigate whether patients with biopsy-proven diagnosis of COVAN have elevated expression of APOL1 protein in their podocytes and GECs, we performed immunohistochemical co-staining of APOL1, synaptopodin (an actin-associated protein of differentiated podocytes), and CD31 (an endothelial cell marker) on kidney biopsy specimens of 2 patients with COVAN diagnosis (Figure 1). APOL1 expression was abundant in glomeruli of patient 1, who underwent biopsy 10 months after COVID-19 diagnosis (Figure 1, A–F), and patient 6, who underwent biopsy 9 days after COVID-19 diagnosis (Figure 1, G–L). In both patients, there was strong APOL1 staining in synaptopodin-positive podocytes (arrow in Figure 1, B, E, H, and K) and along CD31-positive glomerular endothelium (arrowhead in Figure 1, C, F, I, and L). The presence of APOL1 protein in podocytes and GECs at 9 days and at 10 months after diagnosis of COVID-19 infection suggests that APOL1 expression is induced early and may persist in the glomeruli for several months, long after the triggering COVID-19 infection has resolved.

### APOL1 expression is upregulated in biopsy tissue of patients with COVAN but not in control tissue.

**Figure 2:**
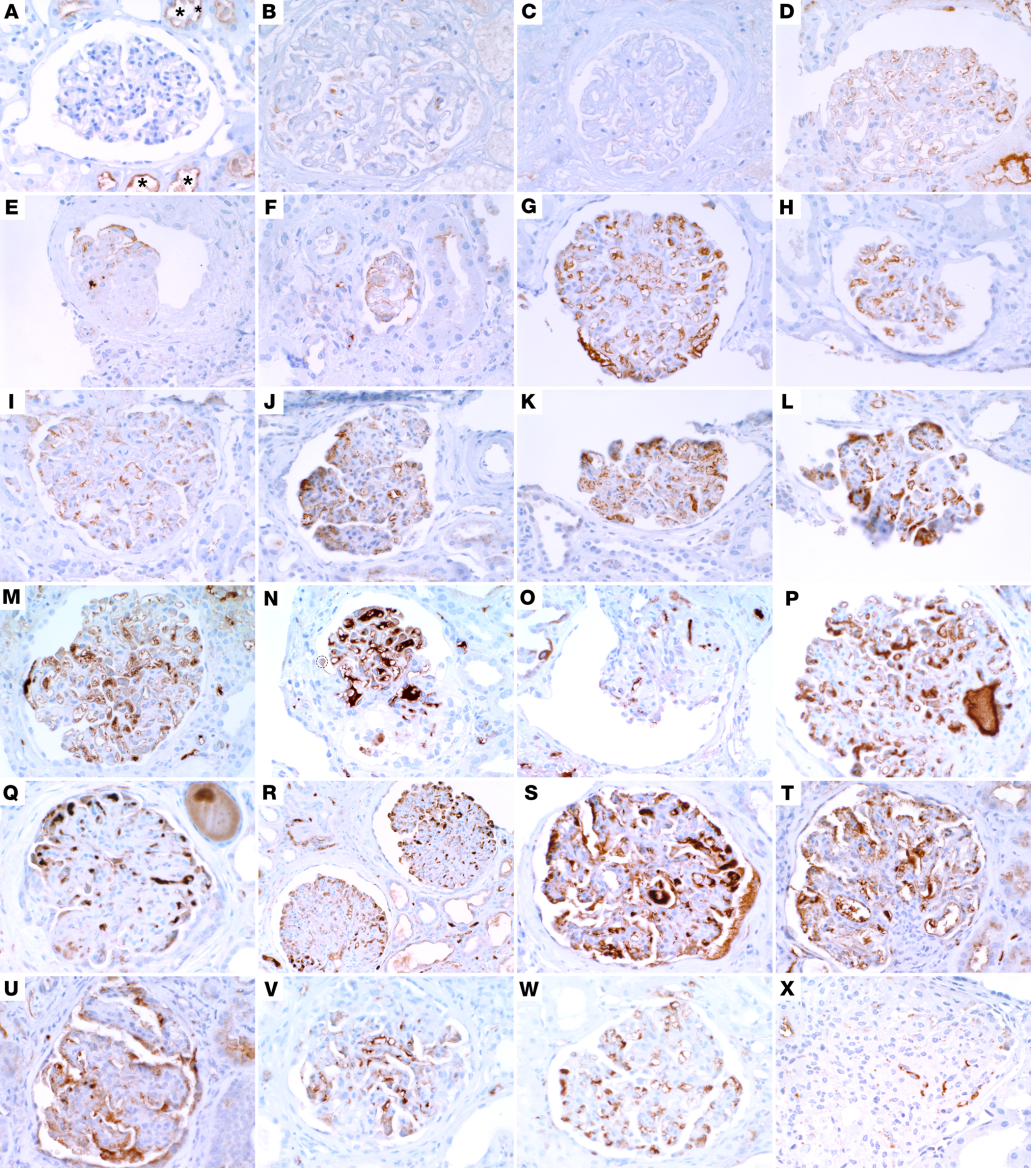
APOL1 expression is upregulated in biopsy tissue of patients with COVAN but not in control tissue. (**A**–**C**) IHC of (**A**) a wedge biopsy specimen from control participant 1 and (**B** and **C**) autopsy specimen from control participant 2 show no APOL1 staining. (**D**–**X**) IHC of APOL1 in (**D**–**F**) patient 1, (**G**–**I**) patient 2, (**J**–**L**) patient 3, (**M**–**O**) patient 5, (**P**–**R**) patient 6, (**S**–**U**) patient 7, (**V**) patient 8, (**W**) patient 9, and (**X**) patient 4. IHC images were taken at the original magnification of 40×, with the exception of **R**, which was taken at the original magnification of 20×.

To further evaluate the generalizability of these immunohistochemical findings, we identified a total of 9 patients with COVAN with available biopsy tissue for genotyping and IHC (Figure 2), as well as 2 control patients, including 1 autopsy control specimen from a patient who had COVID-19 infection but did not develop AKI (Figure 2, B and C). The classic histopathologic features of COVAN included glomerular capillary tuft collapse with adjacent podocyte hypertrophy and proliferation, often with the podocyte protein reabsorption droplets associated with glomerular proteinuria (Supplemental Figures 1 and 2; supplemental material available online with this article; https://doi.org/10.1172/jci.insight.157432DS1). APOL1 IHC staining was absent in all glomeruli of control patients (Figure 2, A–C) but present in glomeruli of all 9 patients with COVAN (Figure 2, D–X). APOL1 staining was abundant in the cytoplasm of podocytes, GECs, and in some parietal epithelial cells (Figures 1 and 2N and Supplemental Figure 3). APOL1 protein could be seen in glomeruli with open capillaries as well as in areas of glomerular collapse. The apparent presence of APOL1 protein within some capillary lumen likely represented circulating APOL1, which is produced primarily by the liver (21). Moreover, APOL1 was also seen in peritubular capillaries and in injured tubular epithelial cells (Figure 2, asterisks). The specificity and significance of this latter finding is unclear. Notably, 7 of the 9 patients with COVAN had a high-risk *APOL1* genotype (Figure 2, D–V). The other 2 patients, patients 9 and case 4, carried a low-risk G0G0 genotype (Figure 2, W and X). APOL1 expression in patient 9, despite having a low-risk genotype, was comparable to those of the 7 high-risk cases (Figure 2W). There were only 2 glomeruli in the slide of patient 4’s kidney biopsy specimen, and APOL1 expression was lower in these glomeruli (Figure 2X). Together, these findings demonstrate that the basal kidney APOL1 expression is low in glomeruli of individuals without glomerular injury, even when the individual has COVID-19 infection; whereas APOL1 expression was upregulated in podocytes and GECs in the setting of COVAN in 89% of patients in this study.

As shown in Table 1, 7 of the 9 patients (77.8%) with biopsy-proven COVAN self-identified as African American. Six of these 7 patients (85.7%) carried a high-risk *APOL1* genotype (4 with G1G1, 1 with G1G2, and 1 with G2G2). By comparison, 13% of Blacks carry a high-risk *APOL1* genotype. The remaining 2 patients self-identified as White Hispanic but, notably, 1 of them also carried a high-risk *APOL1* genotype. In total, 7 of the 9 patients with COVAN (77.8%) carried a high-risk *APOL1* genotype. The median age of the case patients was 51 (range, 37–60) years. All 9 case patients developed AKI and had varying degrees of proteinuria, ranging from subnephrotic to nephrotic range (1.4–14 g/24 hours). Most biopsies were performed at least 1 month after COVID-19 infection, with the exception of patient 6, who underwent biopsy 9 days after a positive PCR test. One patient’s biopsy was not pursued until 10 months after infection secondary to incomplete recovery. The biopsy specimens of all 9 patients exhibited collapsing glomerulopathy, tubular injury, and interstitial inflammation. Endothelial tubular reticular inclusions were not observed in any of the cases. Notably, in the 2 COVAN biopsy specimens that were tested for direct SARS-CoV-2 viral infection by IHC and in situ hybridization, no virus was detected (data not shown).

### Recombinant COVID-19–induced cytokines synergistically upregulate APOL1 expression in primary human GECs and podocytes.

**Figure 3:**
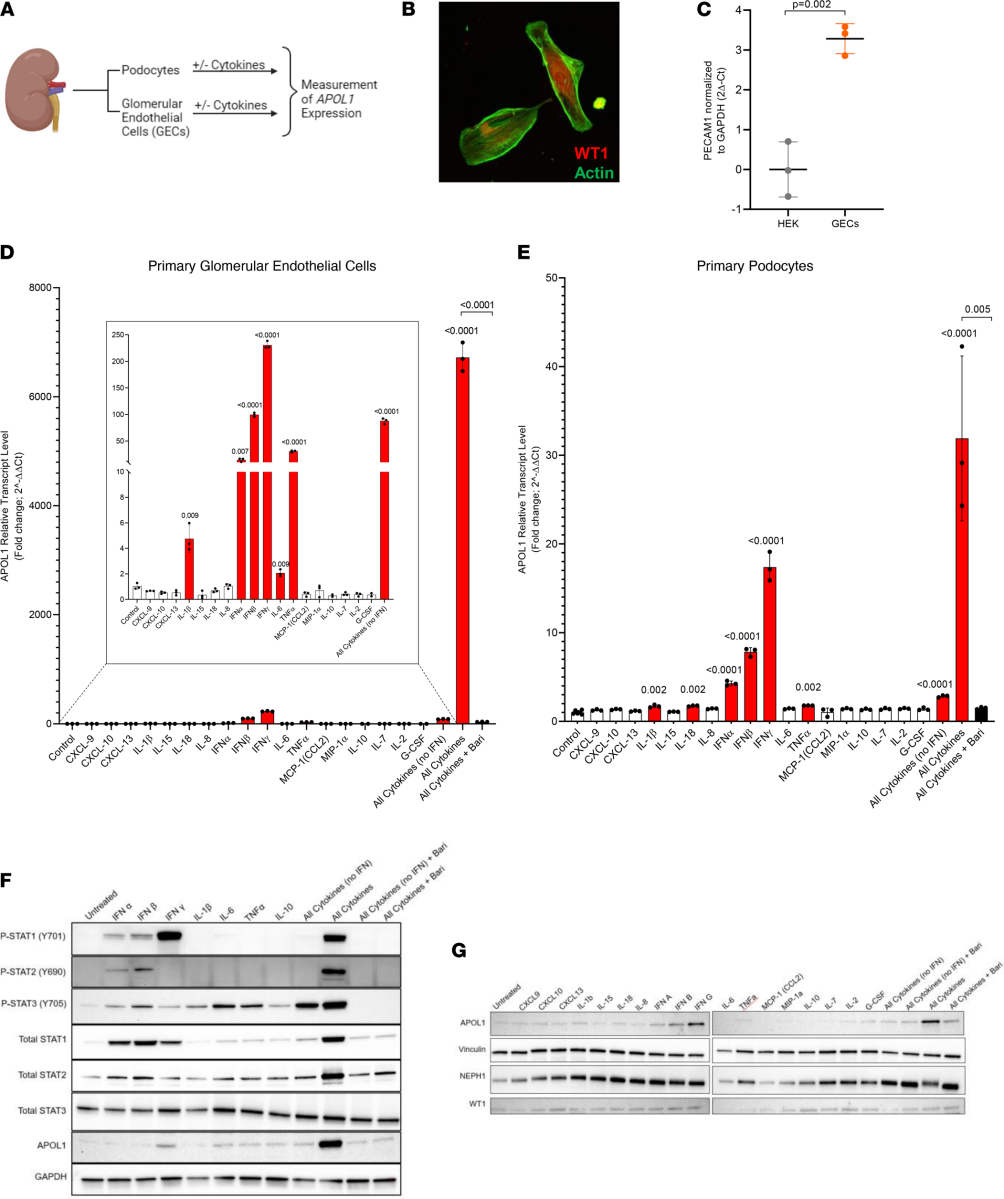
Cytokine storm synergistically induces *APOL1* expression in primary human GECs and podocytes. JAK/STAT signaling mediates COVID-19 cytokine–induced APOL1 expression. (**A**) Experimental design: Primary human podocytes isolated from donor kidney and primary GECs were cultured with or without component cytokines to determine the effect on cellular *APOL1* expression. (**B**) Positive immunofluorescence staining of podocytes for Wilms tumor 1 (podocyte marker). Additional marker staining information is given in Supplemental Figure 4. Original magnification, 20×. (**C**) Quantitative PCR (qPCR) analysis of GECs compared with HEK cells showing enrichment in PECAM1 gene expression (an endothelial marker, also known as CD31). Data are expressed as mean ± SD; *n* = 3. Significance difference assessed by 2-tailed *t* test, with significance set at *P* < 0.05. (**D**) qPCR analysis of *APOL1* mRNA transcript level in GECs and (**E**) podocytes treated for 48 hours with specified individual cytokines, combination of cytokines, and combination of cytokines plus a JAK inhibitor (baricitinib). *GAPDH* was used for normalization. All cytokine concentrations were 50 ng/mL. Cytokine conditions inducing a >1.5-fold *APOL1* transcript compared with media-treated control were further analyzed for significance using unpaired *t* test with Holm-Šidák correction for multiple comparisons. *P* values reported are the adjusted *P* values. Significance was set at *P* < 0.05. Data are expressed as mean ± SD; *n* = 6 for podocyte control and *n* = 3 for all others. Cytokine conditions inducing significantly different *APOL1* expression compared with control are indicated in red. (**F**) WB analysis of APOL1, phosphorylated STAT1–3, and total STAT1–3 in GECs after 48-hour treatment with indicated cytokines, combination of cytokines, and combination of cytokines plus a JAK inhibitor (baricitinib). GAPDH was used as housekeeping protein for comparator. (**G**) WB analysis of APOL1 in podocytes after 48-hour treatment with individual cytokines, combination of cytokines, and combination of cytokines plus baricitinib. Vinculin was used as the housekeeping protein for a comparator. NEPH1 and WT1 are podocyte markers.

To investigate whether COVID-19–induced cytokine storm is sufficient to trigger *APOL1* expression in human glomerular cells, we cultured primary human podocytes isolated from deceased donor kidney and primary human GECs in 18 cytokines and chemokines previously reported to be elevated in the serum of patients with SARS-CoV-2 (Figure 3A) (4, 17). Podocyte identity was confirmed with multiple podocyte markers, including Wilms tumor 1, synaptopodin, nephrin, and podocalyxin (Figure 3B and Supplemental Figure 4, A and B). GEC identity was confirmed by expression of PECAM1 relative to human embryonic kidney 293 cells (Figure 3C). Induced *APOL1* expression was quantitated by quantitative PCR and immunoblot after 48 hours of treatment in GECs (Figure 3, D and F) and podocytes (Figure 3, E and G). Consistent with findings in a prior report (22), we found that IFNs (IFN-γ > IFN-β > IFN-α) robustly induced expression of *APOL1* in GECs and podocytes. Similarly, we found that TNF also induced a modest *APOL1* expression in GECs and podocytes. Unexpectedly, we found that 3 cytokines — IL-6, IL-1β, and IL-18 — which, to our knowledge, were previously unrecognized as inducers of *APOL1* expression, also individually induced modest *APOL1* expression in GECs or podocytes. Notably, the combination of all 18 recombinant cytokines produced a synergistic upregulation of *APOL1* that was an order of magnitude higher than that produced by any of the IFNs alone (Figure 3D). These effects were not only observed with cytokine concentration of 50 ng/mL (Figure 3, D and E) but also at 20 ng/mL and 10 ng/mL (Supplemental Figure 5).

Cytokine conditions inducing greater than a 1.5-fold *APOL1* transcript compared with media-treated control were further analyzed for significance. Significance was assessed (*t* test with Holm-Šidák correction for multiple comparisons and adjusted *P* values).

These results expand the list of physiologic cytokines that can induce *APOL1* expression beyond the well-recognized IFNs and TNF. Importantly, the findings also suggest that the synergy of COVID-19–induced cytokines may be more relevant for *APOL1* regulation than the impact of an isolated cytokine alone.

### JAK/STAT signaling mediates COVID-19-cytokine–induced APOL1 expression.

We next investigated whether COVID-19–induced cytokines upregulate *APOL1* expression via a common intracellular signaling pathway that could be exploited as a therapeutic target. It was previously reported that IFN induction of *APOL1* is mediated by JAK-STAT1/2 (22, 23). Signaling through the IL-6 receptor is mediated by STAT3, and both IL-1β and TNF are reported to indirectly activate STAT3 (24, 25). Of interest, Meliambro et al. (26) recently reported upregulation of phosphorylated STAT3 in the biopsy tissue of a case of COVAN and HIV-associated nephropathy (HIVAN) compared with a control. On the basis of this background information, we hypothesized that the JAK1/2-STAT1/2/3 pathways are the primary mediators of the effects of COVID-19–induced cytokines in driving *APOL1* expression.

To test this hypothesis, we determined the state of these signaling pathways by measuring the phosphorylated STAT1, STAT2, and STAT3 in lysates of GECs after culturing them in individual or combined cytokines for 48 hours (Figure 3F). Type I IFNs (namely, IFN-α and IFN-β) increased phosphorylation of STAT1–3, and IFN-γ upregulated phosphorylation of STAT 1 and STAT3. IL-1β, TNF, and IL-6 increased phosphorylation only of STAT3. Combined cytokines increased phosphorylation of STAT1–3. Knowing that JAK1 and JAK2 are the primary upstream protein kinases that phosphorylate STAT1–3, we hypothesized that inhibition of JAK1/2 would block *APOL1* expression induced by all cytokines. Consistent with this prediction, we found that the JAK1/2-specific inhibitor baricitinib significantly reduced *APOL1* mRNA and APOL1 expression by all-cytokine–treated GECs and primary podocytes (Figure 3, D–G). Together, these results demonstrate that JAK/STAT signaling is the primary pathway that mediates COVID-19 cytokine–induced APOL1 expression.

### COVID-19–induced cytokines are sufficient to drive APOL1 expression in human induced pluripotent stem cell–derived kidney micro-organoids via the JAK/STAT pathway.

**Figure 4:**
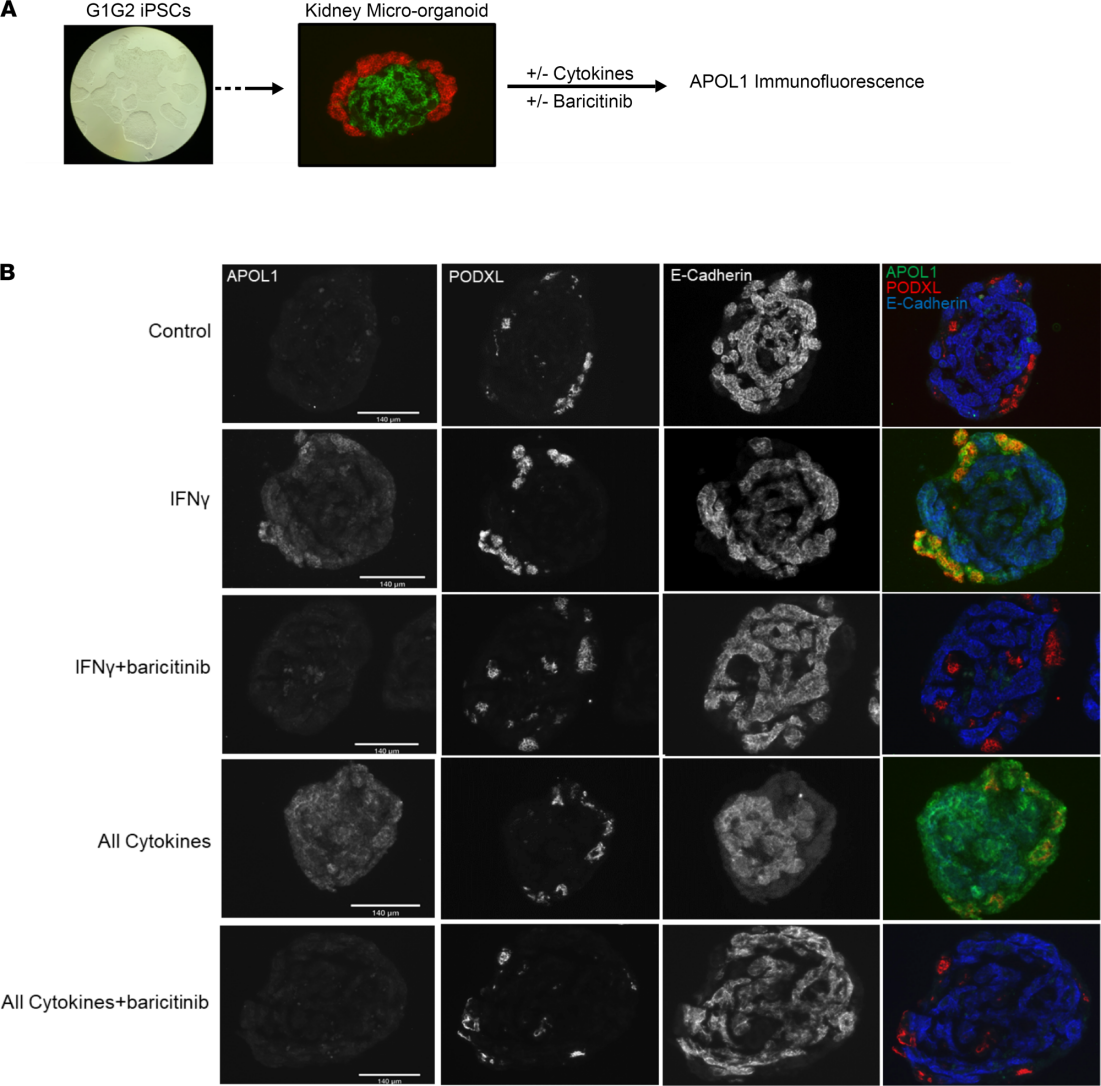
COVID-19–induced cytokines are sufficient to drive APOL1 expression in human iPSC kidney micro-organoids, which is blocked by inhibition of the JAK/STAT/APOL1 axis. (**A**) Experimental design: We created kidney micro-organoids from iPSCs from an individual with G1G2 genotype. Organoids were cultured with or without component cytokines and baricitinib. (**B**) IHC for APOL1, podocyte marker podocalyxin (PODXL), and kidney tubule marker E-cadherin in micro-organoids treated with media alone; IFN-γ alone; IFN-γ plus baricitinib; all cytokines; or all cytokines plus baricitinib. Cytokines were used at a concentration of 10 ng/mL. Baricitinib final concentration was 10 μM. Scale bar: 140 μm.

Human kidney micro-organoid is a proven platform for modeling human kidney disease and facilitating clinical translation. We asked whether the results we obtained from primary human podocytes and GECs were generalizable and could be validated by a human-derived kidney micro-organoid model. Therefore, to investigate APOL1 regulation, expression, and outcomes in this model, we generated kidney micro-organoids from induced pluripotent stem cells (iPSCs) of a Black carrier of the G1G2 *APOL1* genotype (Figure 4A). We cultured kidney micro-organoids in 10 ng/mL IFN-γ or a combination of 8 cytokines (IFN-γ, IFN-α, IFN-β, IL-18, IL-8, IL-6, TNF, and IL-1β) each at 10 ng/mL either in the absence or in the presence of 10 μM baricitinib for 24 hours. These 8 cytokines were chosen because of their observed regulation of *APOL1* expression in the preceding experiments. Podocytes and tubular epithelial cells in the kidney micro-organoids were marker-confirmed (Figure 4B). Consistent with the literature (27), endothelial cells were underrepresented in the kidney micro-organoids (data not shown). We discovered that basal APOL1 protein expression was low in micro-organoids. IFN-γ treatment induced substantial APOL1 expression, with the highest intensity colocalized to areas of the podocyte marker podocalyxin. The cocktail of cytokines induced an outsized and robust APOL1 expression throughout the micro-organoid structure when compared with other treatments. Groups treated with IFN-γ plus baricitinib and all cytokines plus baricitinib showed no APOL1 expression, consistent with complete inhibition of cytokine effect. The APOL1 expression in kidney micro-organoid podocytes was reminiscent of that seen in podocytes of patients with COVAN. However, unlike COVAN kidneys in which no significant APOL1 expression was seen in healthy tubular epithelial cells, kidney micro-organoid E-cadherin–positive tubular epithelial cells expressed APOL1. This difference could be due to differences in membrane cytokine receptors or epigenetic factors that affect protein expression in the immature tubules of the kidney micro-organoids. In summary, human iPSC-derived kidney micro-organoids cultured with COVID-19–induced cytokines showed robust upregulation of pathogenic G1G2 APOL1 protein and the expression was blocked by inhibition of JAK/STAT signaling.

### Cytokine–induced JAK/STAT/APOL1 signaling reduced the viability of kidney micro-organoid podocytes, which was rescued by a JAK inhibitor.

**Figure 5:**
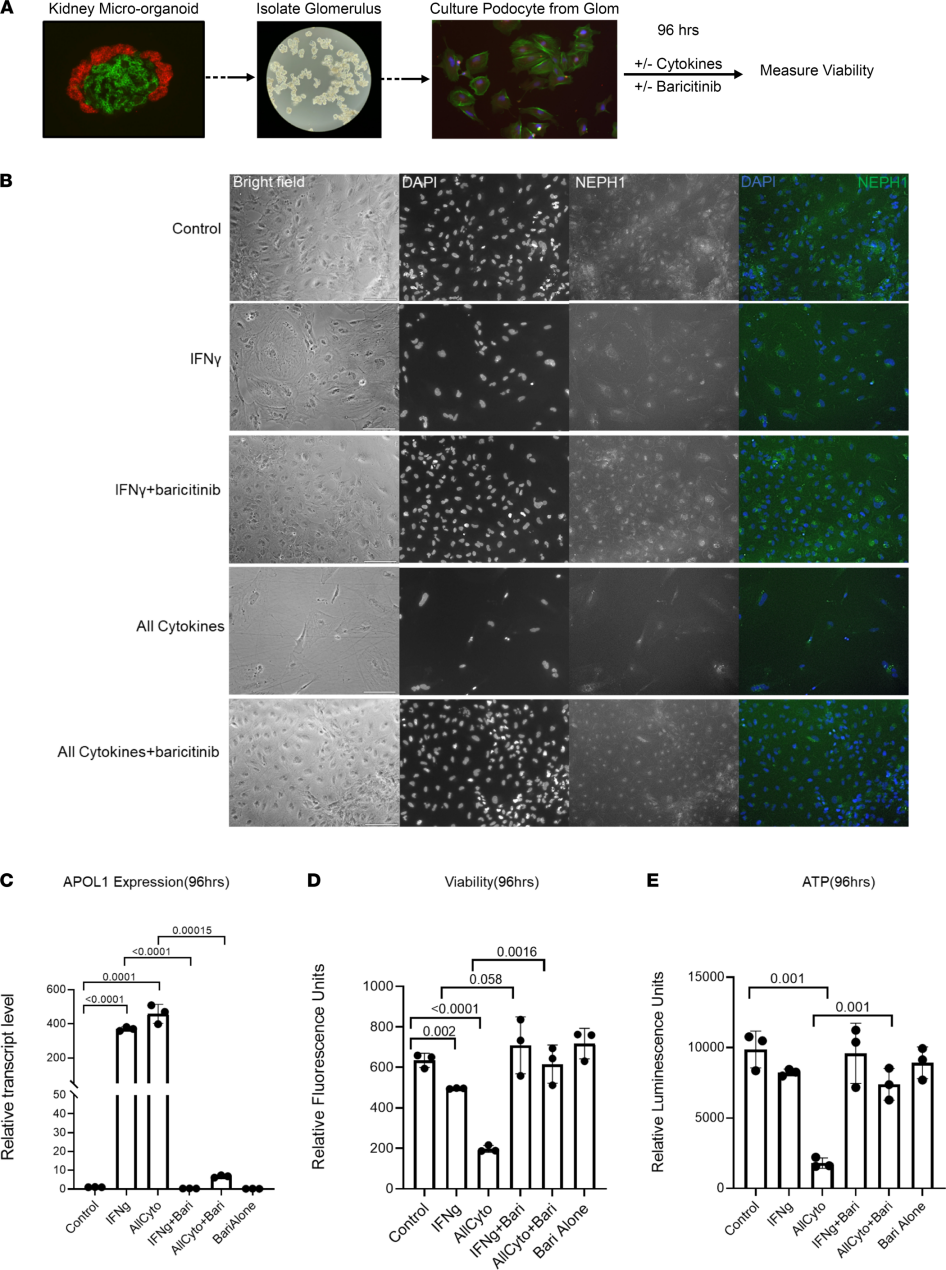
Cytokine-induced *APOL1* expression correlates with significantly decreased viability and cellular metabolism in organoid-derived podocytes (genotype G1G2). Cells are rescued by co-administration with a JAK inhibitor. (**A**) Schemata summarizing the isolation of organoid-derived glomeruli and subculture of podocytes followed by treatment with or without cytokines and baricitinib with subsequent measures in *APOL1*, viability, and IF. (**B**) Representative immunofluorescence staining of DAPI and NEPH1 in organoid-derived podocytes treated for 96 hours with specified treatment conditions; *n* = 4 biological replicates. All cytokine concentrations were 10 ng/mL. Baricitinib at a 10 μM final concentration was used. Scale bar: 140 μm. (**C**) Relative *APOL1* mRNA levels in organoid-derived podocytes treated for 96 hours with IFN-γ (10 ng/mL), combination of cytokines (10 ng/mL), IFN-γ plus JAK inhibitor (baricitinib, 10 μM), and combination of cytokines plus baricitinib (10 μM) versus media-treated control. *GAPDH* was used for normalization. Analysis was performed using a *t* test with significance set at *P* < 0.05. Data are expressed as mean ± SD; *n* = 3. (**D**) Relative fluorescence units were measured after performing a viability assay in organoid-derived podocytes treated for 96 hours with IFN-γ (10 ng/mL), combination of cytokines (10 ng/mL each), IFN-γ plus a JAK inhibitor (baricitinib, 10 μM), and combination of cytokines plus baricitinib (10 μM) versus media-treated control. Data are expressed as mean ± SD. Significance was determined by *t* test with α = 0.05; *n* = 3 representing independent biological replicates. (**E**) Relative luminescence units were measured to quantitate ATP as an indicator of metabolically active cells in organoid-derived podocytes treated for 96 hours with IFN-γ (10 ng/mL), combination of cytokines (10 ng/mL each), IFN-γ plus a JAK inhibitor (baricitinib, 10 μM), and combination of cytokines plus baricitinib (10 μM) versus media-treated control. Data are expressed as mean ± SD. Significance was determined by 2-tailed *t* tests with α = 0.05; *n* = 3 representing independent biological replicates.

Finally, we asked if the G1G2 APOL1 expressed in kidney micro-organoid impairs podocyte viability. We hypothesized that a cytokine-induced variant of the APOL1 protein would cause podocyte loss — a hallmark phenotype of COVAN. To test this hypothesis, we isolated podocytes from kidney micro-organoids generated from iPSCs of a carrier of the G1G2 genotype. The podocytes were cultured in IFN-γ (10 ng/mL) or a combination of 8 cytokines (10 ng/mL each), both in the presence and absence of baricitinib (10 μM) for 96 hours (Figure 5, A and B). Cytokine treatment robustly induced *APOL1* expression, and this expression was blocked by baricitinib, consistent with our earlier experiments (Figure 5C). Concordantly, cytokine treatment caused significant podocyte loss as indicated by viability assay and total cellular ATP (Figure 5, D and E). Remarkably, baricitinib completely rescued the cytokine-induced podocyte loss. Together, these results support the conclusion that COVID-19–induced cytokines trigger JAK/STAT/APOL1 signaling, which, in turn, causes podocyte injury and loss. The protective effect of JAK inhibition on podocyte viability strongly supports this hypothesis.

## Discussion

Our major conclusions from the present study are that several COVID-19–induced cytokines, beyond IFNs, act synergistically via JAK/STAT signaling to drive pathogenic APOL1 expression, resulting in podocyte injury and loss, which are blocked by JAK inhibition. On the basis of a case series, we demonstrate that APOL1 protein is abundantly expressed in podocytes and GECs of patients diagnosed with COVAN but not in the glomeruli of healthy control participants or glomeruli of control participants who were COVID-19 positive but COVAN negative. In 3 experimental models, we demonstrate that recombinant cytokines upregulated in COVID-19 infection are sufficient to drive robust APOL1 expression, and unexpectedly, that the strong synergism produced by a combination of cytokines was mediated predominantly through a common intracellular signaling pathway. Collectively, our experimental evidence strongly supports a causal relationship between cytokine-induced JAK/STAT/APOL1 signaling and in vivo COVAN glomerular phenotype, and supports further investigation into this therapeutic target.

The increased frequency of a high-risk *APOL1* genotype (77.8%) among patients with COVAN that we report here correlates with a recent international, multicenter pathology review whose authors reported high-risk *APOL1* genotype in 91.7% of patients with COVAN (3). Given that the frequency of a high-risk *APOL1* genotype in the Black population is 13% (28), discovering a frequency of 77% to 90% in COVAN is profound and comparable to the 60% to 70% frequency reported in HIVAN (28–31). The existence of the remaining 20% to 30% of patients with COVAN (or HIVAN) who do not carry high-risk *APOL1* genotypes suggests the possibility of an *APOL1*-independent pathomechanism or the possibility that, in some cases, COVID-19–induced supraphysiologic expression of G0 APOL1 may also cause podocytopathy. Parsing these possibilities will require more studies. Nevertheless, we previously demonstrated in HEK cells with a tetracycline-inducible APOL1 expression system that cytotoxicity of *APOL1* is both variant and dose dependent (32, 33). Dose-dependent APOL1 cytotoxicity was also reported in similar cell-based systems (34). Moreover, *APOL1* transgenic mouse models have not only validated the causal link between *APOL1* risk alleles and podocyte injury but have demonstrated that the degree of podocytopathy correlated with *APOL1* expression levels (10, 12–14, 35). Our discovery that 8 of 9 patients with COVAN demonstrated robust glomerular APOL1 expression relative to control individuals and the evidence that expression of endogenous *APOL1* risk alleles causes podocytopathy in human kidney micro-organoids support the causal link between APOL1 and podocytopathy. Conversely, the lack of APOL1 in the glomeruli of COVID-19–positive but AKI-negative G0G0 autopsy control tissue suggests that COVID-19 infection without APOL1 induction is not a sufficient driver of COVAN disease.

Our results shift the current paradigm of IFN-induced APOL1 nephropathy. Several examples in which high IFN states caused collapsing glomerulopathy in carriers of a high-risk *APOL1* genotype led to a paradigm that pointed to IFNs as the chief “second-hit” triggers of APOL1-mediated glomerulopathy (31, 36, 37). This paradigm was further reinforced by the fact that IFN-α, IFN-β, and IFN-γ induced *APOL1* expression in cultured podocyte and endothelial cell lines (22). However, this archetype is challenged by the observation that IFN levels are not always elevated in serum of patients with COVID-19 infection and COVAN, whereas levels of other cytokines, including IL-6, IL-1β, and IL-18, are increased (4, 17, 18). In the present study, we demonstrate that even in the absence of IFNs, these non-IFN cytokines individually and collectively induced robust *APOL1* expression in human podocytes and GECs and highlight a potentially unappreciated synergism. By demonstrating that JAK/STAT signaling is the central mediator of these combined cytokine effects, our results provide a plausible explanation for how the COVID-19 cytokine storm drives *APOL1* expression and the high incidence of collapsing glomerulopathy seen in patients with risk-variant *APOL1* and COVID-19 infection. These findings may have implications for other APOL1-mediated nephropathies.

Although our results show that COVID-19–induced cytokines are sufficient to induce *APOL1* expression and cause the loss of kidney micro-organoid podocytes, they do not exclude the possibility that SARS-CoV-2 may also directly infect kidney cells, as was recently reported (38, 39). However, SARS-CoV-2 in human kidney tissue has only been demonstrated in autopsy specimens in which the confounding contribution of tissue autolysis could not be excluded (3, 4, 15, 16). Most reports from kidney biopsy specimens from patients with COVAN have indicated SARS-CoV-2 virus could not be detected despite the use of sensitive methods (3, 4). These reports include 2 of the 9 patients in our study who were tested by IHC and in situ hybridization.

By demonstrating cytokine-induced podocytopathy, our kidney micro-organoid model diverged from a kidney organoid model of APOL1-mediated kidney disease recently published by Liu et al. (40), who generated kidney organoids from CRISPR-edited iPSCs of a donor of non-African ancestry in which G0 *APOL1* alleles were edited to G1 alleles but on a G0 genetic background. Although IFN-γ induced *APOL1* expression in the Liu et al. organoids, it did not cause cytotoxicity (40). The lack of cytotoxicity in their kidney organoid model could be explained by the less-toxic genetic background on which G1 mutations were superimposed. Cytotoxicity of the *APOL1* haplotype is affected by its genetic background (41). The kidney micro-organoid in the present study was generated from unedited iPSCs of a Black carrier of the G1G2 genotype. Preservation of the native genetic haplotype may have contributed to the APOL1-associated cytotoxicity seen in our kidney micro-organoids.

Furthermore, in contrast to the upregulated APOL1 protein expression we report here, authors of a recent study found no difference in *APOL1* mRNA level in the kidney biopsy specimen of 1 patient with COVAN, compared with healthy control participants (26). This disagreement in our results may be explained by the transient nature of *APOL1* mRNA relative to protein, especially when biopsy specimens were obtained several weeks after the initial diagnosis of COVID-19 infection, when the acute effects of COVID-19–induced cytokine storm and corresponding mRNA expression profile may have dissipated. It is conceivable that the farther one is from the COVID-19–induced cytokine storm, the weaker the acute-phase reactants downstream of the cytokine receptor become, including phosphorylated STAT proteins and *APOL1* mRNA. Our results suggest that the induced APOL1 protein persists beyond *APOL1* mRNA and phosphorylated STATs.

This study has 3 major clinical implications. First, our findings underscore the need to genotype Black or Hispanic individuals with collapsing glomerulopathy in the context of active or recent COVID-19 infection. However, if kidney biopsy is not feasible or possible, *APOL1* genotyping of Black or Hispanic individuals infected with COVID-19 and with new or worsening proteinuria and AKI is also likely to be high yield. Second, because multiple COVID-19–induced cytokines redundantly activate the JAK/STAT pathway to induce *APOL1* expression, a therapeutic strategy based on selective removal or inhibition of any 1 cytokine is unlikely to be effective in preventing or treating COVAN. Currently, baricitinib is only authorized for use under an emergency-use authorization for treatment of COVID-19 requiring supplemental oxygen. Therefore, its potential as a treatment for COVAN requires serious consideration, especially for Black and Hispanic carriers of high-risk *APOL1* genotypes who have COVID-19 infection. Third, on the basis of the evidence that IFN deficiency is associated with severe COVID-19 infection, it was proposed that IFN be administered as therapy for COVID-19. At the time of this writing, according to ClinicalTrials.gov, 36 clinical trials of IFN as therapy in COVID-19 are either ongoing or completed. In contrast to the rationale behind these clinical trials, our results caution against administration of IFNs as treatment for COVID-19 infection in carriers of high-risk *APOL1* genotype, because IFNs could upregulate expression of pathogenic APOL1 in the kidney and precipitate COVAN.

Our study has some limitations. Our COVAN case series of 9 biopsy-proven collapsing glomerulopathy cases is a relatively small sample size and may have underestimated the association between high-risk genotype and COVAN or been subject to sampling bias. The logistical challenge of obtaining kidney biopsy specimens from patients with active COVID-19 infection in the acute care setting limits the frequency of kidney biopsy specimens in this population. The same factor is likely responsible for the fact that most of the biopsies in this study were performed several weeks to months after the initial diagnosis of COVID-19 infection. Analysis of kidney biopsy specimens obtained closer to the infectious trigger may provide additional insights into early cellular phenotypes. Additionally, we do not identify in the present study who and when to treat COVAN with JAK inhibitors. Answers to these questions and the determination of the efficacy of JAK inhibition as treatment for COVAN will be the focus of future investigations.

This work highlights the association of high-risk *APOL1* genotype and the role of JAK/STAT/APOL1 signaling in the development of COVAN. As our understanding of the COVID-19 pandemic evolves, there is an urgent need to increase clinician and public health awareness about the renal complications of COVID-19. There is also an urgent need for COVAN therapy. Our study offers new data on JAK inhibitors as strong therapeutic candidates for APOL1-associated COVAN.

## Methods

### Abs and reagents.

Primary Abs against the following proteins were used: APOL1 rabbit anti-human (Genentech, 3.1C1 and 3.7D6; Western blot [WB] 1:5000 [final concentration 0.05 μg/mL; Genentech, 5.17D12]; IHC 1:4000 [final concentration 0.95 μg/mL], per ref. 42); APOL1 mouse anti-human (Genentech, 4.17A5; immunofluorescence [IF] 1:2000 [final concentration 2.13 μg/mL]); GAPDH mouse anti-human (Santa Cruz Biotechnology, sc47724; WB 1:200); vinculin mouse anti-human (MilliporeSigma, V9131; WB 1:200); NEPH1 mouse anti-human (Santa Cruz Biotechnology, sc373787; WB 1:300); WT1 rabbit anti-human (Abcam, ab89901; WB 1:1000); STAT1 mouse anti-human (Cell Signaling Technology [CST], 9176s; WB 1:1000); STAT2 rabbit anti-human (CST, 72604; WB 1:1000); STAT3 mouse anti-human (CST, 9139; WB 1:1000); phosphorylated STAT1 (Y701) rabbit anti-human (CST, 9167; WB 1:1000); phosphorylated STAT2 (Y690) rabbit anti-human (CST, 88410; WB 1:1000); phosphorylated STAT3 (Y705) rabbit anti-human (CST, 9145; WB 1:2000); PODXL goat anti-human (R&D Systems, AF1658; IF 1:500), E-cadherin rabbit anti-human (CST, 3195S; IF 1:200), and NEPH1 mouse anti-human (Santa Cruz Biotechnology, sc-373787; IF 1:100). Secondary Abs included goat anti–rabbit IgG, HRP-linked Ab (CST, 7074s; WB 1:1000); horse anti–mouse IgG, HRP-linked Ab (CST, 7076s; WB 1:1000); Alexa Fluor 488–conjugated donkey anti-mouse (Jackson ImmunoResearch, 715-546-150; IF 1:1000), Alexa Fluor 594–conjugated donkey anti-goat (Jackson ImmunoResearch, 705-585-147; IF 1:1000), and Alexa Fluor 405–conjugated donkey anti-rabbit (Thermo Fisher Scientific, A48258; 1:000). For real-time quantitative reverse transcription PCR (qRT-PCR), TaqMan gene expression assays included APOL1 (Hs01066280_m1), GAPDH (Hs03929097_g1), and PECAM-1 (Hs00169777_m1) (Thermo Fisher Scientific, catalog 4331182).

### Kidney IHC staining for APOL1, synaptopodin, and CD31.

Starting with FFPE kidney tissue biopsy specimen slides, antigen retrieval was performed with EDTA solution at pH 8.0 for 56 minutes at 100°C. Primary Abs against APOL1 (5.17D12, Genentech), synaptopodin (Progen Biotechnik, 61094), and CD31 (CST, 3528s) were applied at 1:4000 (final concentration 0.95 μg/mL), 1:100, and 1:1600, respectively, for 60 minutes at 36°C. Ready-to-use 3-hydroxy-2-quinoxaline secondary anti-rabbit multimers (catalog 760-4815, Roche) were incubated for 12 minutes at 36°C. This was followed by addition of anti–3-hydroxy-2-quinoxaline HRP for 12 minutes. DAB (catalog 760-159, Roche) was incubated for 5 minutes at room temperature. Tissue section was counterstained hematoxylin (catalog 760-2021, Roche) for 4 minutes at room temperature. Bluing reagent (catalog 760-2037, Roche) was added for 4 minutes at room temperature.

### Cytokine treatment of cultured cells.

Primary human GECs, primary podocytes subcultured from human donor kidney, and organoid-derived podocytes were treated with 50 ng/mL, 20 ng/mL, or 10 ng/mL concentration of the following cytokines: recombinant human CXCL9 (BioLegend, 578102); recombinant human CXCL10 (BioLegend, 573502); recombinant human CXCL13 (BioLegend, 573502); IL-1β (PeproTech, 200-01B); IL-15 (PeproTech, 200-15); IL-18 (BioLegend, 592102); IL-8 (BioLegend, 574202); IFN-α1 (MilliporeSigma, SRP4596); IFN-β (PeproTech, 300-02BC); IFN-γ (MilliporeSigma, I17001); IL-6 (PeproTech, 200-06); TNF-α (PeproTech, 300-01A); MCP-1 (CCL2; PeproTech, 300-04); MIP-1α (CCL3; PeproTech, 300-08); IL-10 (PeproTech, 200-10); IL-7 (PeproTech, 200-07); IL-2 (PeproTech, 200-02); and G-CSF (PeproTech, 300-23). A standardized cytokine concentration of 50 ng/mL was predetermined on the basis of precedent from human cell treatments evaluating cytokine-shock syndromes in COVID-19 infection (17). Follow-up experiments used concentrations of 20 ng/mL and 10 ng/mL. Subsequent treatments, including organoid and organoid-derived podocyte experiments, were performed using a cytokine concentration of 10 ng/mL. The JAK1/2 inhibitor baricitinib (INCB028050; Selleckchem, S2851) was used at 10 μM final concentration in all experiments.

### Primary human GEC culture.

Frozen stock of low-risk (G0G0) primary human GECs was purchased from Celprogen (36066-05), thawed, and cultured in human glomerular endothelial primary cell culture complete medium with serum, antibiotic free (Celprogen, M36066-05SA), on plates coated with a proprietary extracellular matrix (Celprogen, E36066-05-PD10 and E36066-05-12Well) in a humidified environment at 37°C and 5% CO_2_. Cells were passaged using 1× trypsin EDTA (Celprogen, T1509-014). Cells were used for experiments between passages 2 and 4. Cells were validated by qRT-PCR showing enrichment in PECAM1 gene expression (endothelial cell marker, also known as CD31).

### Glomerular isolation from donor human kidney and podocyte subculture.

The human donor kidney was procured through the National Disease Research Interchange. The donor APOL1 genotype was G0G1. Glomerular isolation was performed using the sieve method adapted from prior publications (43, 44). Briefly, working on ice in a sterile hood, the kidney was first decapsulated and cut in half mid-sagittally. The medulla was dissected away, leaving the cortex. The cortex was then minced and passed sequentially through stainless steel mesh sieves (sizes 425 μm and 250 μm) and collected on the top of a third sieve (150 μm) while washing frequently with precooled PBS with 1% BSA (without calcium and magnesium; Endecotts, sieves 100SIW.150, 100SIW.250, and 100SIW.425). Glomeruli were collected, centrifuged, and resuspended in 5 mL PBS. Then, 10 μL of sample was stained with NucBlue Live ReadyProbes Reagent (Hoechst 33342; Thermo Fisher Scientific, R37605) and visualized by light microscopy. Glomeruli were then incubated in digestion buffer (DMEM/F12; 1 mg/mL each of collagenase I, IV, and V; DNase I [50 U/mL or 50 μg/mL]) at 37°C for 1 hour to obtain a single-cell suspension (Thermo Fisher Scientific, 10565018; Stemcell Technologies, 07415, 07426, 07430; MilliporeSigma, 11284932001; respectively). DMEM/F12 with 10% FBS (R&D Systems, S10350H) was added to stop digestion, and the sample was centrifuged at 450*g* for 5 minutes at 4°C. Isolated podocytes were subcultured in Advanced RPMI medium (Thermo Fisher Scientific, MT10040CV) with 10% FBS and 1% penicillin-streptomycin (Thermo Fisher Scientific, 15070063) on vitronectin-coated plates (Stemcell Technologies, 07004) at 37°C and 5% CO_2_. Subcultured cells were visible at approximately 5 days and were treated after 2 weeks in culture.

### Protein extraction and WB.

All culture plates and samples were maintained at 4°C. Monolayers of cells were lysed and harvested with Cell Lysis Buffer (CST, 9803) with complete miniprotease inhibitor and phosphatase inhibitor (MilliporeSigma, 04693159001, 04906837001). Samples were sonicated at level 4 for 10 seconds each, centrifuged at 13.5*g* for 5 minutes at 22°C, supernatant was collected in a new Eppendorf tube, and protein concentration was determined by BCA protein assay (Pierce, 23225). Protein lysates were diluted with 4× LaemmLi sample buffer plus 2-mercaptoethanol (Bio-Rad, 1610747; Thermo Fisher Scientific, 21985023; respectively) and heated at 95°C to 100°C for 5 minutes. Protein lysates were then separated using Criterion TGX stain-free gels (4%–20%) and transferred using Bio-Rad Trans-Blot Turbo transfer system. Transferred membranes were blocked for 1 hour in 3% nonfat milk in Tris-buffered saline and incubated with specific primary Abs overnight at 4°C. On the next day after standard washing, membranes were incubated with HRP-conjugated secondary Ab for a minimum of 1 hour at room temperature prior to imaging with a Bio-Rad ChemiDoc MP Imaging System per manufacturer’s instructions.

### RNA extraction and qRT-PCR.

RNA was isolated from cell monolayers using RLT lysis buffer (Qiagen) and the Qiagen RNeasy Mini Kit (catalog 74106) per manufacturer’s instructions. RNA was transcribed into cDNA using Invitrogen SuperScript IV Reverse Transcriptase reagents and protocol (Thermo Fisher Scientific, 18091050). qRT-PCR reactions were performed with Applied Biosystems’ TaqMan Fast Advanced Master Mix (Thermo Fisher Scientific, 44-445-57) and gene expression assays using QuantStudio 6 Flex System (Thermo Fisher Scientific) using the ΔΔC_t_ method with GAPDH as the reference gene.

### Genotyping.

DNA was extracted from FFPE tissue blocks using the QIAamp DNA FFPE Tissue Kit (Qiagen, catalog 56404) per manufacturer’s protocol. Genotyping was performed using Applied Biosystems’ TaqMan allelic discrimination assays for G1 SNP (p.Ser342Gly) and G2 polymorphism (p.Asn388_Tyr389del) using the QuantStudio 6 Flex System. This assay has 100% analytic specificity and an analytic sensitivity (limit of detection) of 1.0 ng of DNA for the detection of *APOL1* risk variants in DNA extracted from peripheral blood monocytes. Assays were previously validated by comparing results with those of direct sequencing (Sanger sequencing). Genotyping quality control measures included the use of technical replicates, 100% matching of positive controls of each genotype, and negative controls.

### Derivation of G1G2 patient iPSC kidney micro-organoid.

Kidney micro-organoids were derived per published protocol (27), with some modifications. Briefly, human iPSCs were dissociated into single cells using TrypLE Select and seeded onto vitronectin-coated plates in StemFlex medium (Thermo Fisher Scientific, A3349401) with 10 μM Rho kinase inhibitor (Tocris Bioscience, 1254) at a density of 11,000 to 14,000 cells/cm^2^ and cultured in a humidified environment at 37°C and 5% CO_2_. Cells were then transitioned to TeSR-E6 medium (Stemcell Technologies, 05946) with 8 μM CHIR99021 (Tocris Bioscience, 4423) for 4 days. From day 5 to day 7, cells were treated with 200 ng/mL FGF9, 1 μg/mL heparin, and 1 μM CHIR99021. On day 7, cells were washed with PBS and dissociated with TrypLE. Dissociated cells were then washed with plain DMEM and centrifuged at 300*g* for 5 minutes. The cell pellet was resuspended in stage 1 medium (TeSR-E6 containing 200 ng/mL FGF9, 1 μg/mL heparin, 1 μM CHIR99021, 0.1% poly[vinyl alcohol] [PVA], 10 μM Rho kinase inhibitor [Tocris Bioscience]) and transferred to a 24-well AggreWell 400 plate (Stemcell Technologies, 34411) at approximately 1.2 million cells/well. The plate was then centrifuged at 100*g* for 3 minutes and incubated for 48 hours at 37°C and 5% CO_2_ in a standard cell-culture incubator. After 48 hours (day 9), organoids from the AggreWell 400 plate were transferred to a 6-well low-attachment plate with stage 2 medium (TeSR-E6 containing 200 ng/mL FGF9, 1 μg/mL heparin, 1 μM CHIR99021, 0.1% poly[vinyl alcohol]) on an orbital shaker inside a cell incubator for another 72 hours. From day 12 onward, all organoids were refreshed with stage 3 medium (TeSR-E6 containing 0.1% poly[vinyl alcohol]) on alternative days until used for experiments.

### Podocyte isolation from micro-organoids.

Isolation of glomeruli from kidney organoids was adapted from a previously described protocol (45). Briefly, groups of iPSC-derived kidney organoids with an initial starting cell number of 1.2 × 10^6^ iPSCs were dissociated by incubation with TrypLE select (Thermo Fisher Scientific) for 5 minutes at 37°C. Gentle mixing using a 1 mL pipette was applied every 2 to 3 minutes to aid dissociation. Per group, a single 70 μm cell strainer (pluriSelect) was placed onto a 50 mL tube (Falcon, Corning), and the mesh was hydrated with PBS. The cell solution was added to the mesh using a 1 mL pipette, allowing flow through of the solution by gravity. Using the plunger from a 1 mL sterile syringe, the remaining cell solution captured on the strainer was gently pushed through the mesh. The strainer was washed with PBS and discarded, keeping the cell flow-through. The cell flow-through was then pipetted onto a prehydrated 40 μm cell strainer (pluriSelect) placed onto a fresh 50 mL tube (Falcon, Corning), allowing single cells to flow through by gravity and by washing the sieve extensively with PBS to remove any remaining single cells. The largest glomeruli were then collected from the 40 μm cell strainer by inverting the sieve onto a fresh 50 mL tube and washed using PBS to flush out the captured glomeruli. This process was repeated using flow-through from the 40 μm process using the final 30 μm cell strainer (PluriSelect) to collect the smaller glomeruli. Isolated glomeruli from iPSC-derived kidney organoids were cultured on vitronectin-coated plates with Advanced RPMI containing 10% FBS in a standard cell-culture incubator at 37°C plus 5% CO_2_. The media were refreshed every other day until cells were used for experiments.

### IF staining: micro-organoids.

Kidney micro-organoids were washed with PBS and fixed in 4% paraformaldehyde (PFA) in PBS for 30 to 45 minutes on ice. Fixed organoids were then washed 3 times with PBS and incubated in 30% sucrose in PBS overnight at 4°C. Organoids were embedded in Tissue-Tek O.C.T. Compound (Sakura Finetek) and subjected to 10 μm cryosectioning with a Leica cryostat. Organoid cryosections were washed 3 times with PBS and then blocked with blocking buffer 1 (1% fish gelatin, 2% donkey serum, 0.3% Triton X-100 in PBS) for 1 hour at room temperature. Next, cryosections were incubated with primary Abs in blocking buffer 1 overnight at 4°C. Cryosections were washed 3 times with PBS. After washing, cryosections were incubated with secondary Abs in blocking buffer 1 for 2 hours at room temperature. After 5 washes with PBS, the cryosections were mounted with ProLong Glass Antifade Mounting Solution (Thermo Fisher Scientific, P36980). Fluorescence images were generated using an Echo Revolve-M26 microscope.

### IF staining: podocytes.

Podocyte cultures were washed with PBS and fixed in 4% PFA in PBS for 10 to 15 minutes at room temperature. After fixation, cells were washed 3 times with PBS and blocked in blocking buffer 2 (1% fish gelatin, 2% donkey serum, 0.1% saponin in PBS) for 30 to 60 minutes. Cells were incubated with primary Abs in blocking buffer 2 for 2 hours at room temperature (or overnight at 4°C). Cells were washed 3 times with blocking buffer 2 and incubated with secondary Abs in blocking buffer 2 for 1 hour at room temperature. After 3 washes with blocking buffer 2 and 1 final wash with PBS, the cells were mounted with Drop-n-Stain EverBrite mounting medium (Biotium, 23008). Fluorescence images were captured using an Echo microscope.

### Viability testing.

For cell viability and ATP measurements, organoid-derived podocytes were plated onto a vitronectin-coated 96-well plate in 100 μL of medium (Stemcell Technologies, 07004; Corning, CLS3603) and cultured in Advanced RPMI+10%FBS+1%penicillin-streptomycin (Thermo Fisher Scientific, MT10040CV; Thermo Fisher Scientific, 15070063) in humidified environment at 37°C and 5% CO_2_. Organoid-derived podocytes were simultaneously plated onto a 6-well plate to be used for RNA extraction for qPCR and onto a 24-well plate to be used for IHC. Cells were treated at approximately 80% confluence with 6 conditions (control; IFN-γ 10 ng/mL; IFN-γ 10 ng/mL + baricitinib [final concentration 10 μM]; all cytokines 10 ng/mL; and all cytokines 10 ng/mL + baricitinib [final concentration 10 μM]). Media were changed every 48 hours, and cells were evaluated at 96 hours. The 96-well plate was processed using Promega cell viability and ATP assays as discussed below; the 6-well plate was processed for RNA extraction, cDNA synthesis, and qRT-PCR; and the 24-well plate was fixed in 4% PFA for IHC. CellTiter-Fluor Cell Viability Assay (Promega, G6080) and CellTiter-Glo 2.0 Assay (Promega, G9241) were performed using standard protocol instructions provided by manufacturer. The assays were multiplexed per protocol. Fluorescence (nonlytic protease assay) and luminescence (lytic ATP assay) were measured using SpectramaxM3 fluorometer. Difference in measures was determined by Student’s unpaired *t* test.

### Statistics.

All data are presented as mean ± SD. GraphPad Prism 8.3.1 software was used for data analysis. Cytokine conditions inducing greater than 1.5-fold *APOL1* transcript compared with medium-treated control were analyzed for significance using an unpaired 2-tailed *t* test with Holm-Šidák correction for multiple comparisons. *P* values reported are the adjusted *P* values. Significance was set at *P* < 0.05.

### Study approval.

This study was approved by the IRB of Duke University, North Carolina. Patient-informed consent was not required by the IRB, because the case portion of this study was a retrospective review of clinical and archived pathologic material only. Human kidney for glomerular isolation and podocyte subculture was procured through the National Disease Research Interchange (NDRI); this human tissue research was also preapproved through Duke’s IRB. NDRI requires all tissue-source sites to obtain informed consent from the tissue donor or surrogate.

## Author contributions

SEN, GL, SD, KS, and DS performed experiments and edited the manuscript. AW and DBT performed and interpreted histopathology and IHC, and edited the manuscript. GH contributed essential reagents and edited manuscript. SEN and OAO designed the study, analyzed and interpreted data, designed figures, and wrote and edited the manuscript.

## Supplementary Material

Supplemental data

## Figures and Tables

**Table 1:**
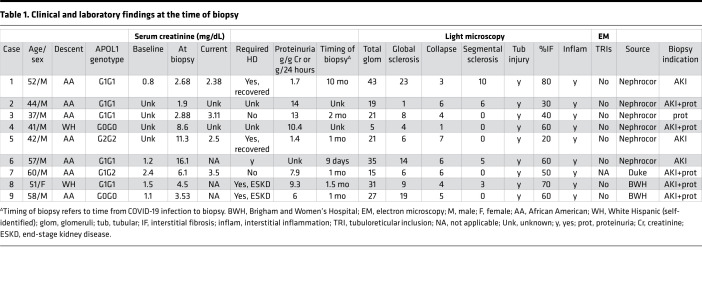
Clinical and laboratory findings at the time of biopsy

## Version 1 04/26/2022

In-Press Preview

## Version 2 06/08/2022

Electronic publication

## Version 3 06/09/2022

Graphical Abstract added
