# Supplementary material for: JAK inhibitor blocks COVID-19 cytokine–induced JAK/STAT/APOL1 signaling in glomerular cells and podocytopathy in human kidney organoids
Source: JCI Insight. 2022 Jun 8;7(11):e157432. doi: 10.1172/jci.insight.157432 (PMC9220952; doi:10.1172/jci.insight.157432)
Supplement: Supplemental data [file jciinsight-7-157432-s288.pdf]

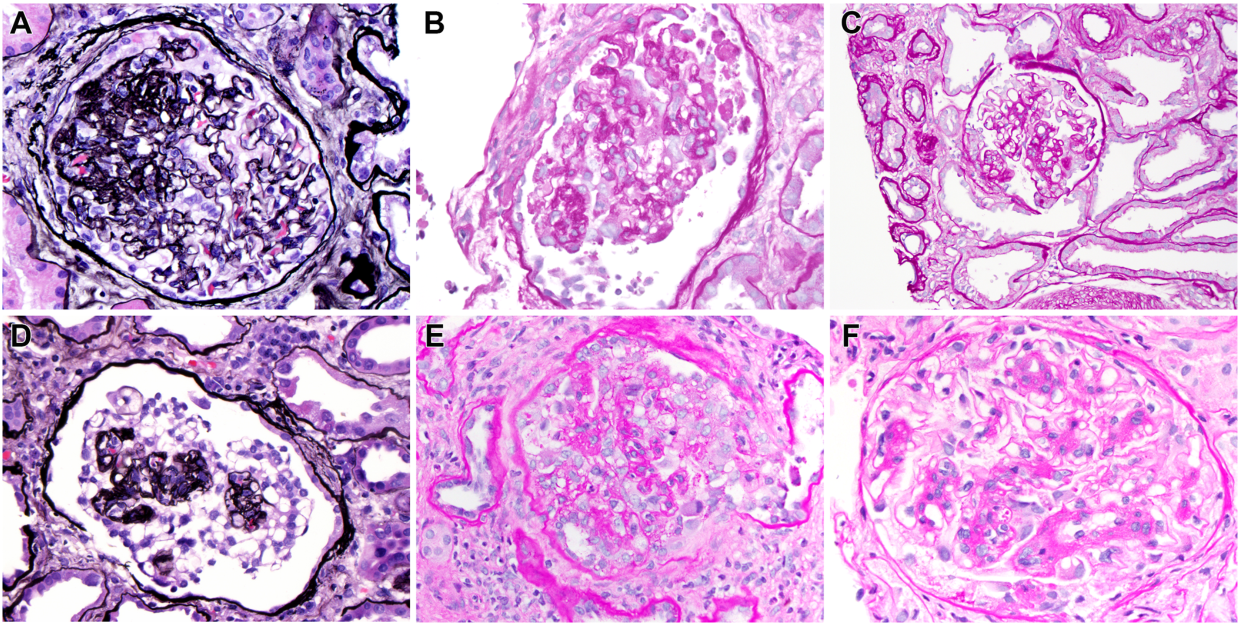

Supplemental Figure 1

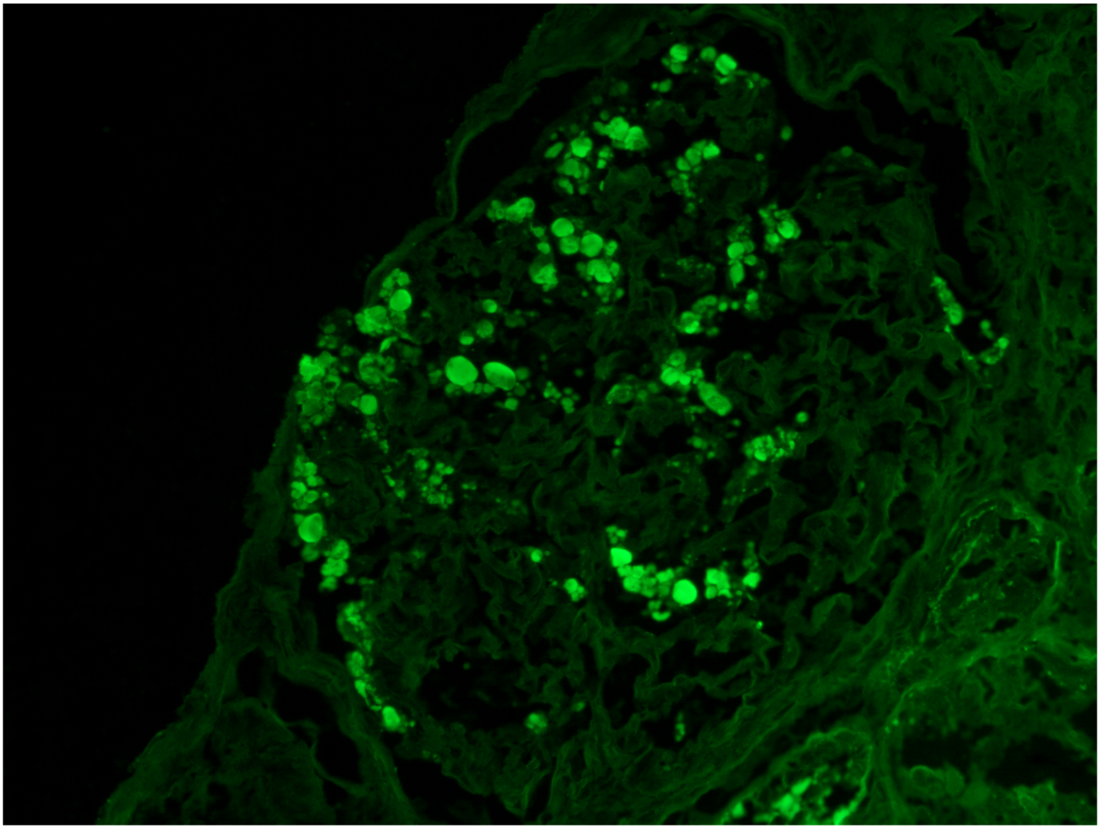

Podocyte protein reabsorption droplets (albumin immunofluorescence), Case 2

Supplemental Figure 2

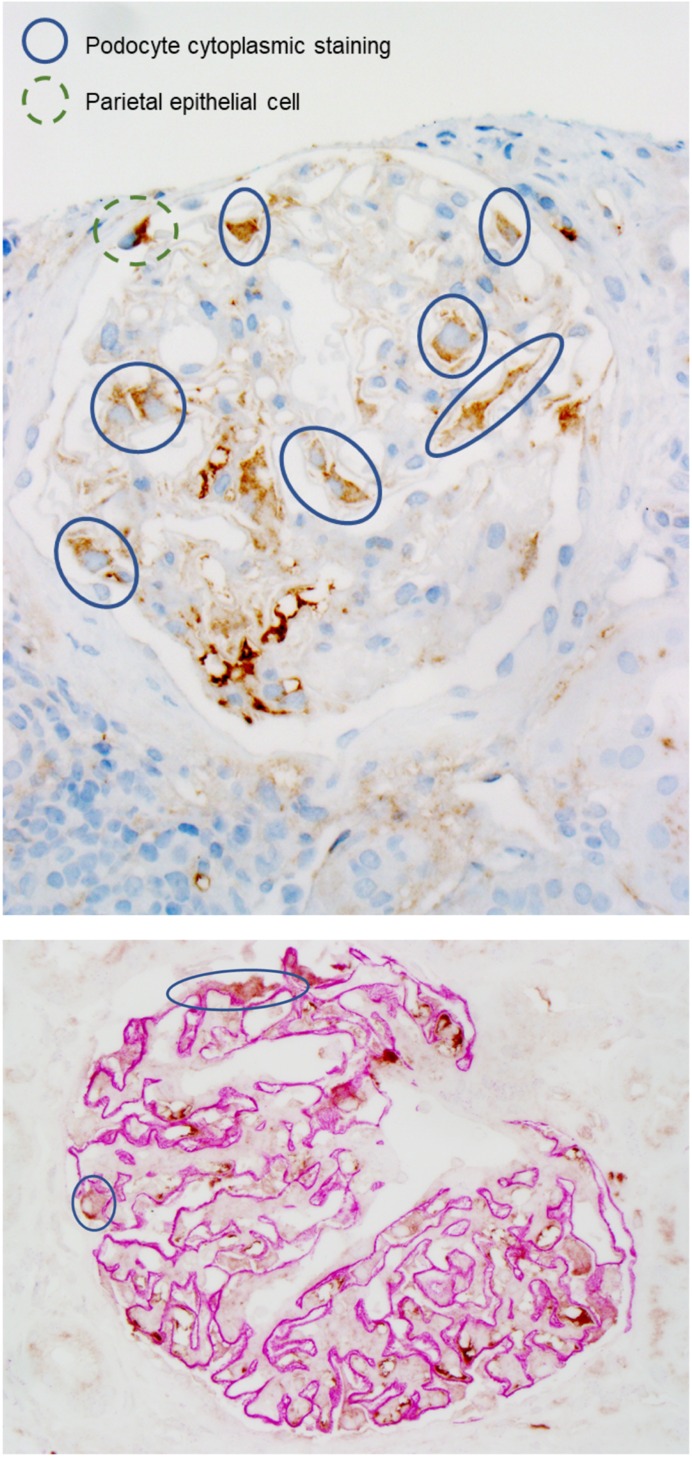

Supplemental Figure 3

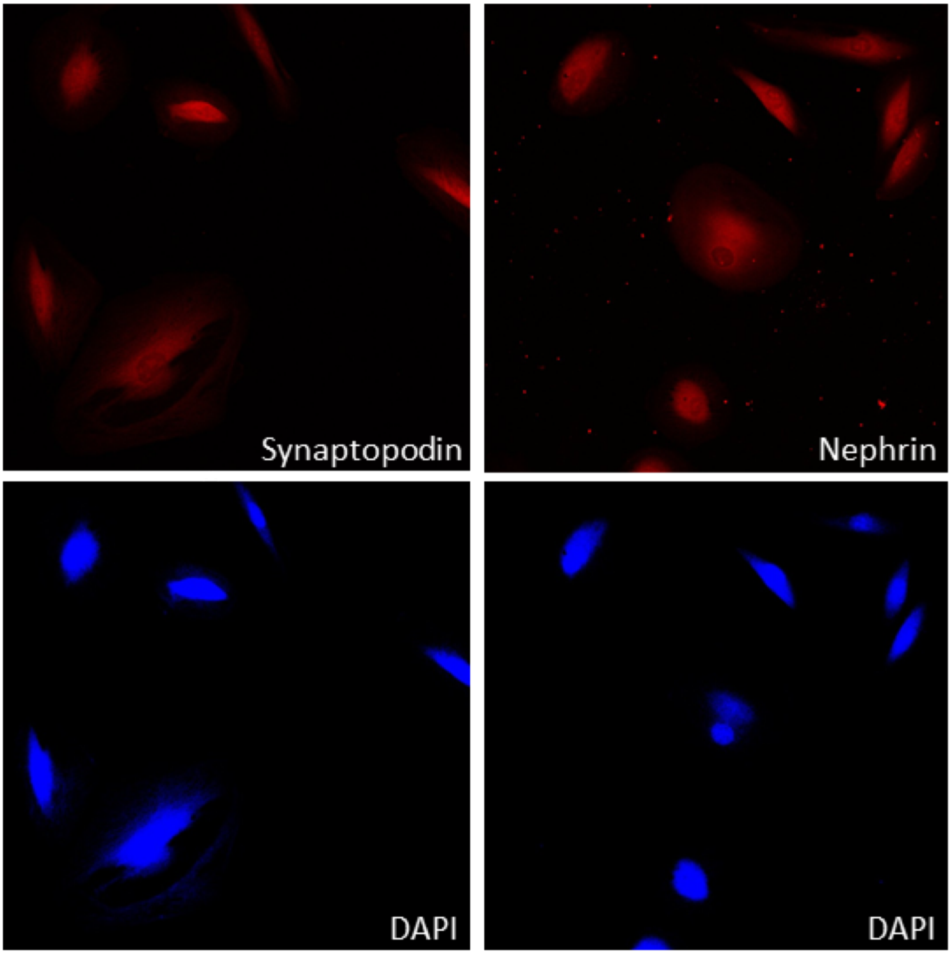

Supplemental Figure 4A

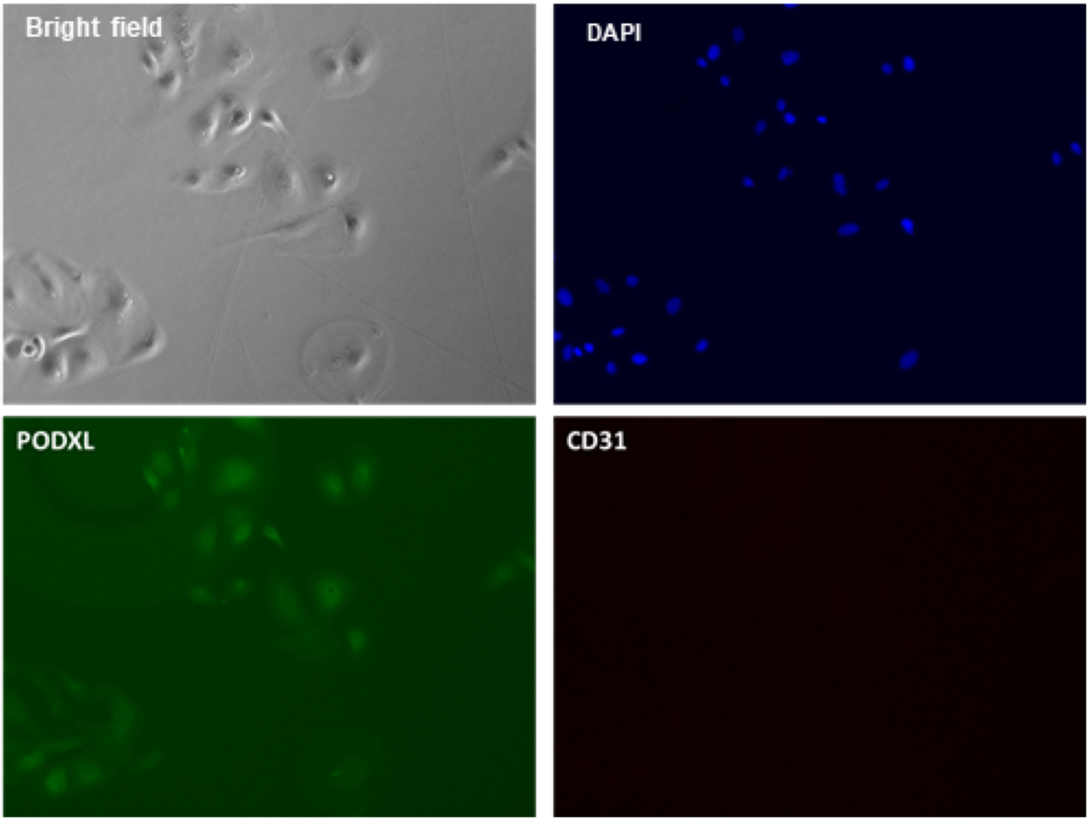

Supplemental Figure 4B

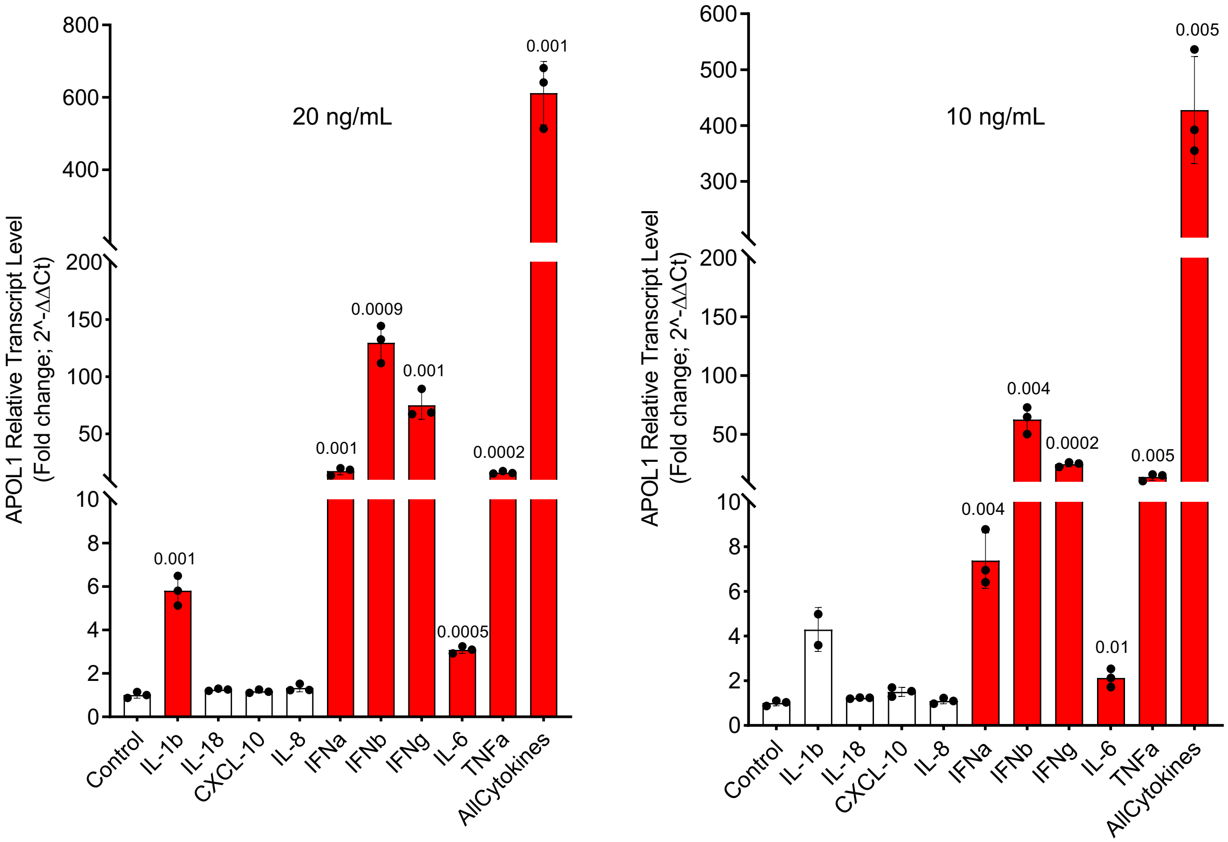

Supplemental Figure 5

Supplemental Figure Legend

Supplemental Figure 1. COVID-19 associated collapsing glomerulopathy (COVAN) with capillary tuft wrinkling, retraction with collapse, and adjacent epithelial cell hypertrophy and proliferation.

A. Case 1, Jones methenamine silver stain
B. Case 2, Periodic acid-Schiff
C. Case 4, Periodic acid-Schiff
D. Case 5, Jones methenamine silver stain
E. Case 8, Periodic acid-Schiff
F. Case 9, Periodic acid-Schiff

Supplemental Figure 2. Podocyte protein reabsorption droplets shown by albumin immunofluorescence in Case 2.

Supplemental Figure 3. IHC of APOL1 (shown in brown color) for Case 1. APOL1 staining can be seen in the podocyt cytoplasm (blue circles) and in parietal epithelial cell (green dashed circle). Purple staining in right panel is synaptopodin.

Supplemental Figure 4. Primary human podocyte identity confirmed by immunofluorescent staining.

A. Positive immunofluorescent staining of podocyte markers (synaptopodin, nephrin, and Wilms tumor 1) in primary human podocytes.
B. Positive immunofluorescent staining of podocyte marker (podocalyxin) and negative staining for endothelial cell marker (CD31 or PECAM-1).

Supplemental Figure 5. Quantitative real-time PCR analysis of *APOL1* mRNA transcript level in GECs treated x48hrs with specified individual cytokines and combination of cytokines at 20ng/mL and 10ng/mL concentrations compared to media-treated controls. *GAPDH* was used for normalization. Cytokine conditions inducing >1.5 fold *APOL1* transcript compared to media-treated control were further analyzed for significance using unpaired t-test with Holm-Sidak correction for multiple comparisons. P-values reported are the adjusted p-values. Significance was set at $p<0.05$. Data are expressed as mean +/-SD, n=3. Cytokine conditions inducing significantly different *APOL1* expression compared to control are indicated in red.
